# In Vivo Assessment of Cortical Bone Fragility

**DOI:** 10.1007/s11914-020-00558-7

**Published:** 2020-02-22

**Authors:** Lyn Bowman, Anne B. Loucks

**Affiliations:** 1 Department of Biological Sciences and the Ohio Musculoskeletal and Neurological Institute, Ohio University, Athens, OH 45701 USA; 2 AEIOU Scientific, LLC, Ohio University, Athens, OH 45701 USA

**Keywords:** Fracture, Cortical bone, Bone mechanics, Bending test, Bone stiffness, Bone strength

## Abstract

**Purpose of Review:**

This review updates readers on recent developments in the assessment of cortical bone fragility in vivo. The review explains the clinical need that motivated the development of Cortical Bone Mechanics Technology™ (CBMT) as a scientific instrument, its unique capabilities, and its necessary further development as a medical device.

**Recent Findings:**

Clinical experience with dual-energy X-ray absorptiometry has led to calls for new clinical methods for assessing bone health. CBMT is a noninvasive, dynamic 3-point bending test that makes direct, functional measurements of the mechanical properties of cortical bone in ulnas of living people. Its technical validity in accurate measurements of ulna flexural rigidity and its clinical validity in accurate estimations of quasistatic ulna bending strength have been demonstrated.

**Summary:**

Because CBMT is a whole bone test, its measurements reflect the influences of bone quantity and bone quality at all hierarchical levels.

## Introduction

More than 40 years ago, quasistatic, single load-to-failure tests of whole long bones ex vivo demonstrated that bending stiffness is a very accurate predictor of the maximum load a bone can bear before breaking [1, 2]. Cortical Bone Mechanics Technology™ (CBMT) performs a *dynamic* 3-point bending test to make direct, noninvasive measurements of ulna bending stiffness in humans in vivo. Recent quasistatic validation of these measurements has confirmed that they also predict ulna bending strength very accurately [3•]*.*

Because CBMT is a whole bone test, its measurements reflect the sum total of all factors operating at all hierarchical levels of the ulna, from whole bone geometry to tissue material properties and composition, porosity, microarchitecture, and nanoscale collagen cross-linking and protein-mineral bonding [4]. Thus, *CBMT captures the influences of bone quality as well as bone quantity on the ulna’s load-bearing capacity, however, that may have been affected by genetics, modeling, remodeling, nutrition, activity, aging, disease, pharmacological intervention,* etc.

We describe here our motivation for developing and commercializing CBMT, its unusual capabilities, and its remaining unknowns.

### The Clinical Need: Fewer Errors in Allocating Patients to Treatment

T-scores of areal bone mineral density (aBMD) measured by dual-energy X-ray absorptiometry (DXA) perform well in their original epidemiological application [5]. They identify a *subpopulation* of patients (those with aBMD T-scores < −2.5) with a higher risk of fracture than the rest. It is in their subsequent clinical application for allocating *individuals* to treatment to prevent fractures that aBMD T-scores have disappointed expectations by failing to predict fractures well [6]. As will be shown below, most patients diagnosed with osteoporosis in epidemiological studies have not fractured, and most fractures have occurred among patients who did not have osteoporosis.

In the field of machine learning, the performance of a method for binary classification of a population can be assessed by a type of contingency table known as a confusion matrix [7].

**Fig. 1:**
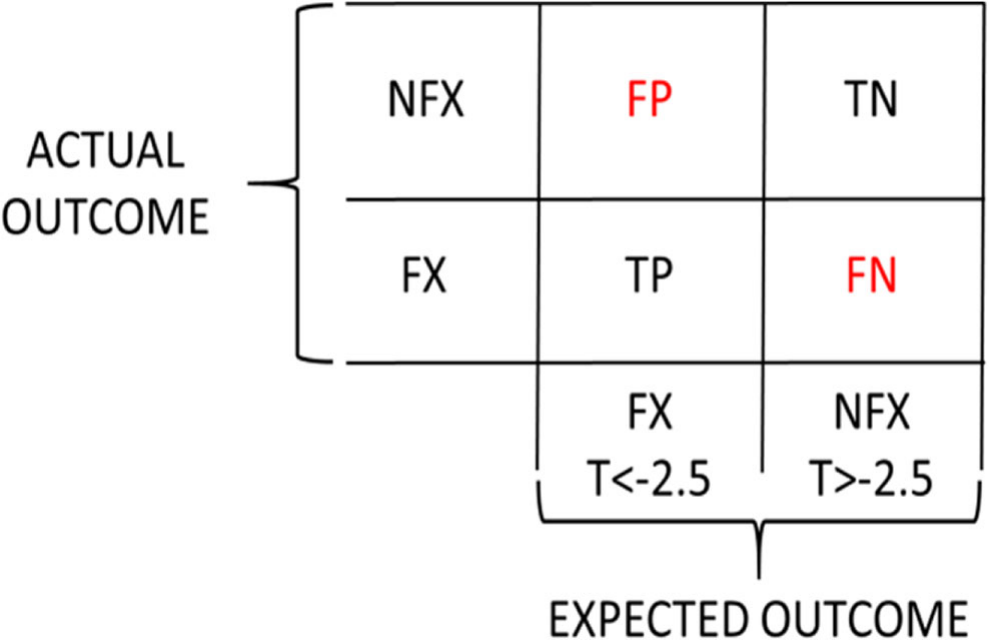
The confusion matrix for using aBMD T-scores for allocating patients to treatment to prevent fractures. TP = True Positive, FP = False Positive, FN = False Negative, TN = True Negative

Figure 1 shows the confusion matrix for binary assignment of patients to treatment by aBMD T-scores. Patients expected to fracture (FX T < −2.5) are assigned to treatment, while treatment is withheld from those not expected to fracture (NFX T > −2.5). Actual outcomes differ. Some patients allocated to treatment do fracture (TP = True Positive), but others do not (FP = False Positive), and some patients from whom treatment is withheld do not fracture (TN = True Negative), while others do (FN = False Negative).

**Fig. 2:**
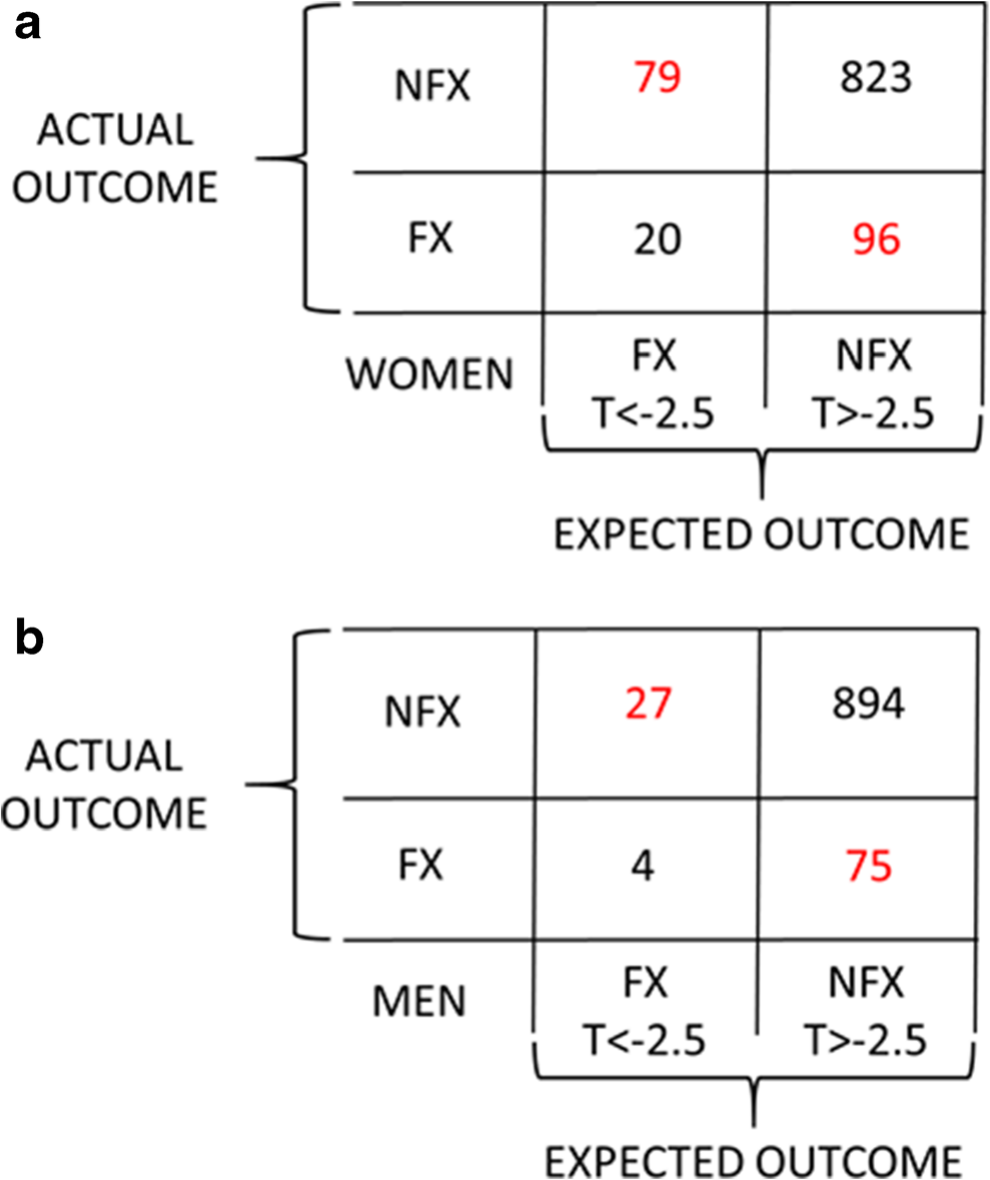
The confusion matrix for allocations of 1000 women (**a**) and 1000 men (**b**) to treatment based on T-scores of aBMD. FX = fracture, NFX = no fracture, FX T < −2.5 = fracture expected, NFX T > −2.5 = fracture not expected. (Data from [8•])

Results of a recent meta-analysis [8•] of femoral neck aBMD T-scores from 7254 women and men in eight studies of fractures are summarized in the confusion matrix shown in Fig. 2. For clarity numbers have been normalized to 1000 participants. The risk of fracture in those eligible for treatment (T_aBMD_ < −2.5) was about double the risk in those who were not (T_aBMD_ > −2.5):

$$ {\displaystyle \begin{array}{c}\mathrm{RR}=\left[\mathrm{TP}/\left(\mathrm{TP}+\mathrm{FP}\right)\right]/\left[\mathrm{FN}/\left(\mathrm{FN}+\mathrm{TN}\right)\right]\\ {}\kern0ex {\mathrm{RR}}_{\mathrm{Women}}=\left[20/\left(20+79\right)\right]/\left[67/\left(96+823\right)\right]=1.9\\ {}\kern0ex {\mathrm{RR}}_{\mathrm{Men}}=\left[4/\left(4+27\right)\right]/\left[75/\left(75+894\right)\right]=2.1\end{array}} $$

In a confusion matrix, the statistics that quantify misallocations of individual patients to treatment are the false positive rate (FPR) and false negative rate (FNR). Figure 2 shows that most women eligible for treatment did not fracture (FPR =80%), and most fractures occurred in women who were not eligible (FNR = 83%). Results for men were even worse (FPR = 86%, FNR = 95%).

$$ {\displaystyle \begin{array}{c}\mathrm{FPR}=\mathrm{FP}/\left(\mathrm{FP}+\mathrm{TP}\right)\\ {}\kern0ex \mathrm{FNR}=\mathrm{FN}/\left(\mathrm{FN}+\mathrm{TP}\right)\\ {}\kern0ex {\mathrm{FPR}}_{\mathrm{Women}}=100\left[79/\left(79+20\right)\right]=80\%\\ {}\kern0ex {\mathrm{FNR}}_{\mathrm{Women}}=100\left[96/\left(96+20\right)\right]=83\%\\ {}\kern0ex {\mathrm{FPR}}_{\mathrm{Men}}=100\left[27/\left(27+4\right)\right]=86\%\\ {}\kern0ex {\mathrm{FNR}}_{\mathrm{Men}}=100\left[75/\left(75+4\right)\right]=95\%\end{array}} $$

These high FNR observations seem inexplicable by anything other than misallocation of treatment. However, might the high FPR observations be the happy results of effective fracture preventive care correctly targeted at the patients who need it most? Unfortunately, it would seem not. Of the eight studies included in Fig. 2, two (MrOS in Sweden and Strambo in France) observed only men. Focusing on the high FPR in women in the other six studies, one (Qualyor in France) excluded participants taking osteoporosis medications. Investigators in two of the other five studies responded to our inquiries with information on the use of osteoporosis medications by their participants. In the CaMOS study in Canada, 39% of participants with aBMD T-scores < −2.5 had taken osteoporosis medications [9], and in the GERICO study in Switzerland, only 19% of such participants had done so [10]. In the latter study, most of the osteoporosis medication was in the form of menopausal hormonal therapy taken for reasons other than skeletal health, as it was by similar proportions of participants in osteopenic and normal ranges of aBMD T-scores. Therefore, information from three of the six studies of women indicates that the use of osteoporosis medication does not account for the high FPR in women diagnosed with osteoporosis. And so, we are thrown back on misallocation of treatment as the most likely explanation for the high FPR as well as the high FNR values observed.

*FPR and FNR are important because they are the major drivers of fracture health-care costs.* FP (Type 1) errors drive up cost by administering preventive care to patients who do not need it; and FN (Type 2) errors drive up cost by withholding preventive care from patients who *do* need it and then require treatment for fracture repair. Increasing attention to these disappointing observations [11, 12] has led to widening agreement that “new methods for assessing bone health are required, beyond characterization of mineral density” [4, 13]. *We suggest that FPR and FNR be the metrics by which such new methods are judged.*

### What to Do?

A fracture is a *mechanical* failure of a bone, and the strength of a bone is the maximum load that the bone can bear before breaking. A bone can be weakened prior to fracture by the accumulation of microdamage through cyclic fatigue [14], but still the load that breaks a fatigued bone is its strength in its weakened state.

A caveat is warranted before proceeding. A bone does not have a single strength: the maximum load that a bone can bear depends on whether it is loaded in tension, compression, torsion, shear, bending, or in some combination of these modes. Moreover, because long bones are not axisymmetric, their strength in bending and shear depends upon the direction in which they are loaded, e.g., antero-posteriorly or medio-laterally. As will be shown below, the strength of a bone also depends strongly on whether it is loaded quasistatically (i.e., very, very slowly) as in the widely accepted mechanical single load-to-failure test or dynamically (i.e., rapidly) as in the falls and collisions that occur in real life.

That said, what explains the observations summarized in Fig. 2? The answer to this question is important, because it guides strategy for efforts to improve observations of future clinical outcomes. Two contending hypotheses have been offered.

### The Weak Bone Hypothesis

The Weak Bone Hypothesis holds that people suffer fractures because their bones are weak. By this hypothesis, Fig. 2 infers that aBMD T-scores must not estimate bone strength accurately. This leads to the perceived need for a new clinical method for estimating bone strength more accurately, so that treatment can be targeted at the correct patients: i.e., at FN instead of FP.

**Fig. 3:**
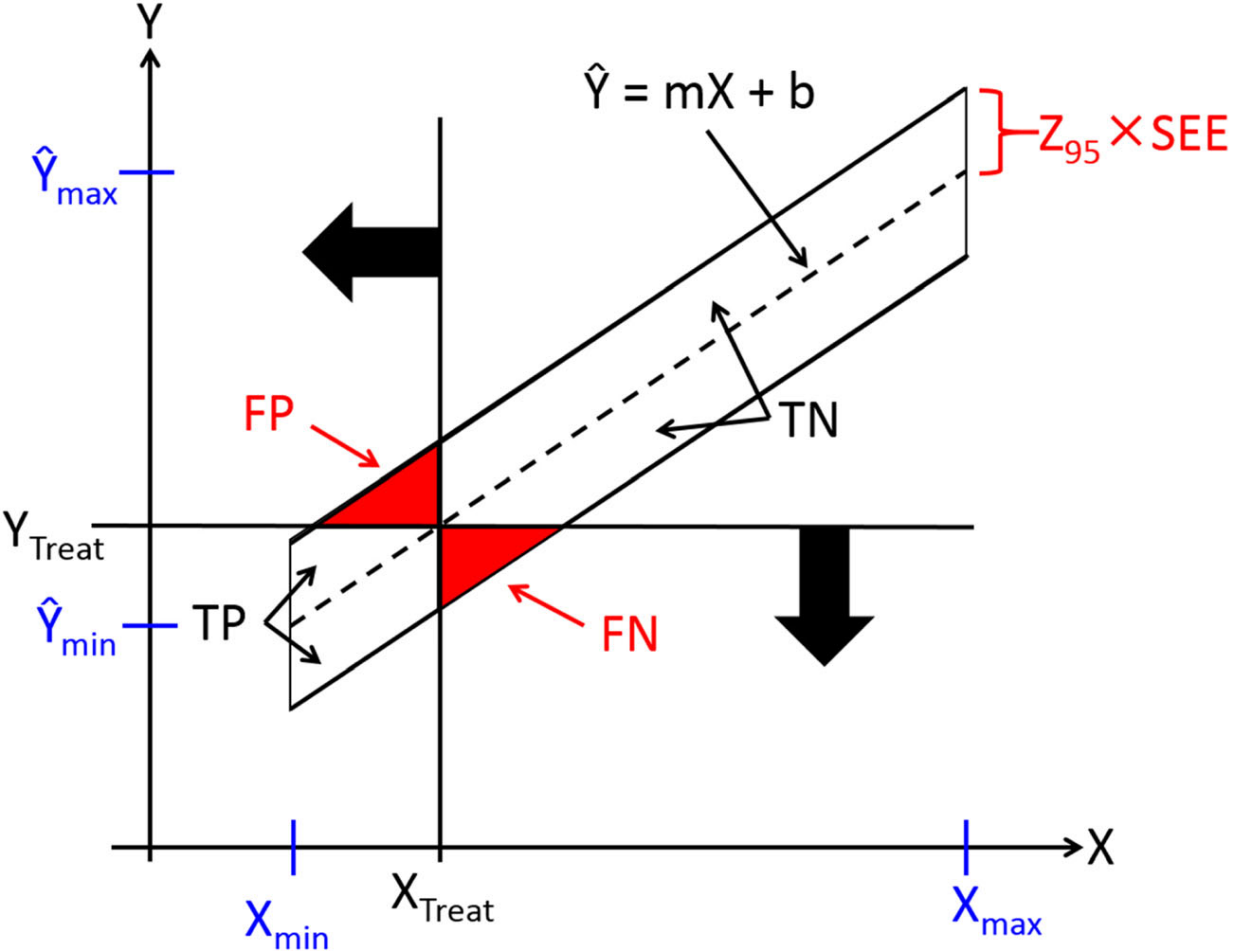
Errors in the treatment of patients below a threshold value (X_Treat_) of a bone strength (Y) predictor X. (TP = True Positive, FP = False Positive, FN = False Negative, TN = True Negative)

Measuring bone strength directly is not a clinical option, because a bone must be broken in order to learn how strong it was before it broke. So, for treatment decisions, an indirect method is required to measure something else that is correlated with bone strength. This predicament is illustrated in Fig. 3 in which one (or conceivably some combination) of the many bone strengths that might be measured appears on the Y-axis, and one (or some combination) of the many correlated predictors of bone strength appears on the X-axis [15].

If the Weak Bone Hypothesis is true, treatment should be administered to patients who fall below some threshold value of Y, Y_Treat_. Instead, treatment is administered to patients who fall below the associated threshold value of X, X_Treat_. At present, X_Treat_ = T_aBMD_ = −2.5.

In Fig. 3, the relationship between Y and X is described by the diagonal regression line Ŷ = mX + b, where m is the slope and b is the Y-intercept of the regression line, and by the standard error of the estimate, SEE, which quantifies the scatter of data points from individual patients around the regression line. Because one of the assumptions of regression analysis is that data points are dispersed uniformly across the entire range of X, the bounds of 95% of the population are represented in Fig. 3 by a pair of lines parallel to the regression line and separated from it by ± Z_95_ × SEE, where Z is the standardized normal variable and Z_95_ = 1.96. (Note that these *parallel* bounds of 95% of the *population* are not the *curved* bounds of the *confidence intervals* on the location of the regression line.) The vertical height of the data cloud (± Z_95_ × SEE) is determined by all the systematic and random sources of error inherent in using X to estimate Y. For aBMD T-scores, the systematic sources of error include everything DXA does not measure, i.e., bone quality.

In Fig. 3, the binary horizontal and vertical treatment decision lines intersecting at the point (X_Treat_, Y_Treat_) and the diagonal bounds of the population discriminate between the True and False Positive and the True and False Negative measures of bone strength and allocations of patients to treatment. Notice that the quadrant form of Fig. 3 corresponds to that of Figs. 1 and 2.

Through elementary geometry and algebra, one can quickly derive from Fig. 3 that for 95% of the population, the total error rate [TER = (FP + FN)/ALL] in allocating patients to treatment is:

$$ \mathrm{TER}=\frac{Z_{95} SEE}{2\left({\hat{Y}}_{max}-{\hat{Y}}_{min}\right)}\approx \frac{SEE}{{\hat{Y}}_{max}-{\hat{Y}}_{min}} $$

TER can be readily calculated by visual inspection of graphs in publications of the mechanical validation of various methods. We will return to this below. For now, notice one last thing about Fig. 3: consistent with the Weak Bone Hypothesis, the strength (Y) of FN is less than that of FP.

Others have explained the observations in Fig. 2 by arguing that treatment decisions for fracture prevention have been based on the wrong property of the wrong type of bone at the wrong skeletal sites [16]. Since most fractures occur at non-vertebral, non-hip sites of predominantly cortical bone in the appendicular skeleton, aBMD T-scores at sites of predominantly trabecular bone in the axial skeleton cannot be expected to predict fractures well. Moreover, since cortical porosity increases with age, treatment decisions need to be guided by a measure of cortical porosity. In addition, since the resistance to bending of cortical bone in the long bones of the appendicular skeleton increases with the fourth power of their diameter, a method is needed for assessing the cross-sectional size of cortical bone. It will be noticed that these criticisms, as well as those pertaining to other neglected aspects of bone quality, all presume the Weak Bone Hypothesis and bear only upon the accuracy with which bone strength is assessed.

Despite the intuitive appeal of the Weak Bone Hypothesis, there is a plausible reason to doubt it, and that reason is classic in the history of science: The Weak Bone Hypothesis fails the parsimony test. The observations in Fig. 2 can be explained without it.

### The Heavy Load Hypothesis

**Fig. 4:**
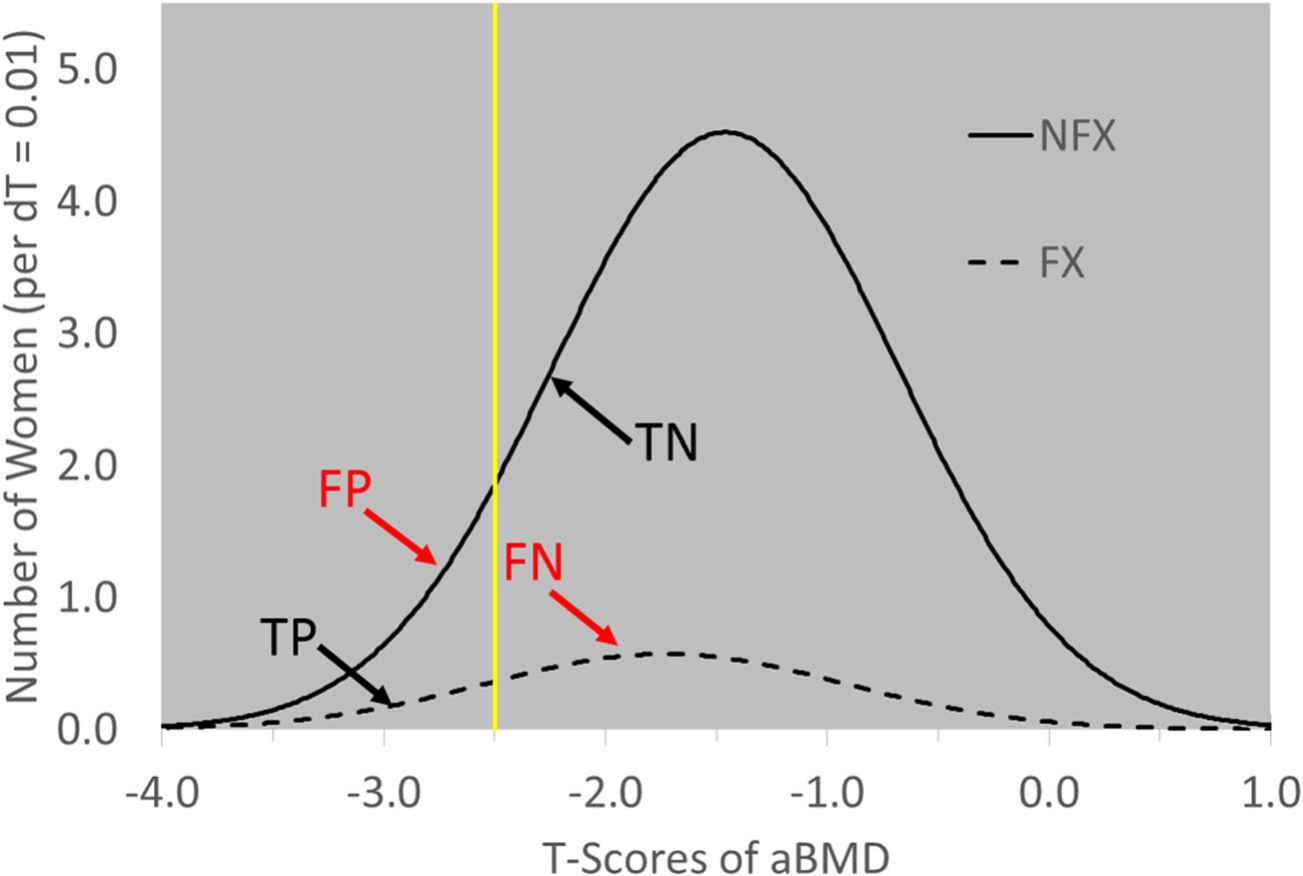
Observed distributions of women with and without fractures allocated to treatment by the threshold T_aBMD_ = −2.5. (FX = women with fractures, NFX = women without fractures, TP = True Positive, FP = False Positive, FN = False Negative, TN = True Negative, dT = increment of T-score)

The Heavy Load Hypothesis holds that people who suffer fractures are exposed to excessively heavy loads. Assuming the subpopulations of women who did and did not fracture in the studies summarized in Fig. 2 (left) were normally distributed on aBMD T-scores, these distributions can be reconstructed from the reported proportions of women with osteoporotic (T < −2.5), osteopenic (−2.5 < T < −1), and normal (T > −1) T-scores in the studies. In Fig. 4, the proportion of women with T_aBMD_ < −2.5 who fractured is RR = 1.9 times the proportion of women with T_aBMD_ > −2.5, as in Fig. 2. In Fig. 4, too, 80% of women allocated to treatment are not fractured (FP in T_aBMD_ < −2.5), and 83% of fractures are in women who did not qualify for treatment (FN in T_aBMD_ < −2.5). *Clearly, these high error rates occur because the distributions of FX and NFX overlap broadly across the range of aBMD T-scores.*

Of course, regardless of how much two distributions overlap, the Central Limit Theorem states that in studies of *groups*, there is a sample size large enough to enable a difference in T_aBMD_ to be detected between FX and NFX through statistical hypothesis testing. *The Central Limit Theorem does not apply to observations of individuals, however, and so it does not help physicians make treatment decisions for individual patients.*

**Fig. 5:**
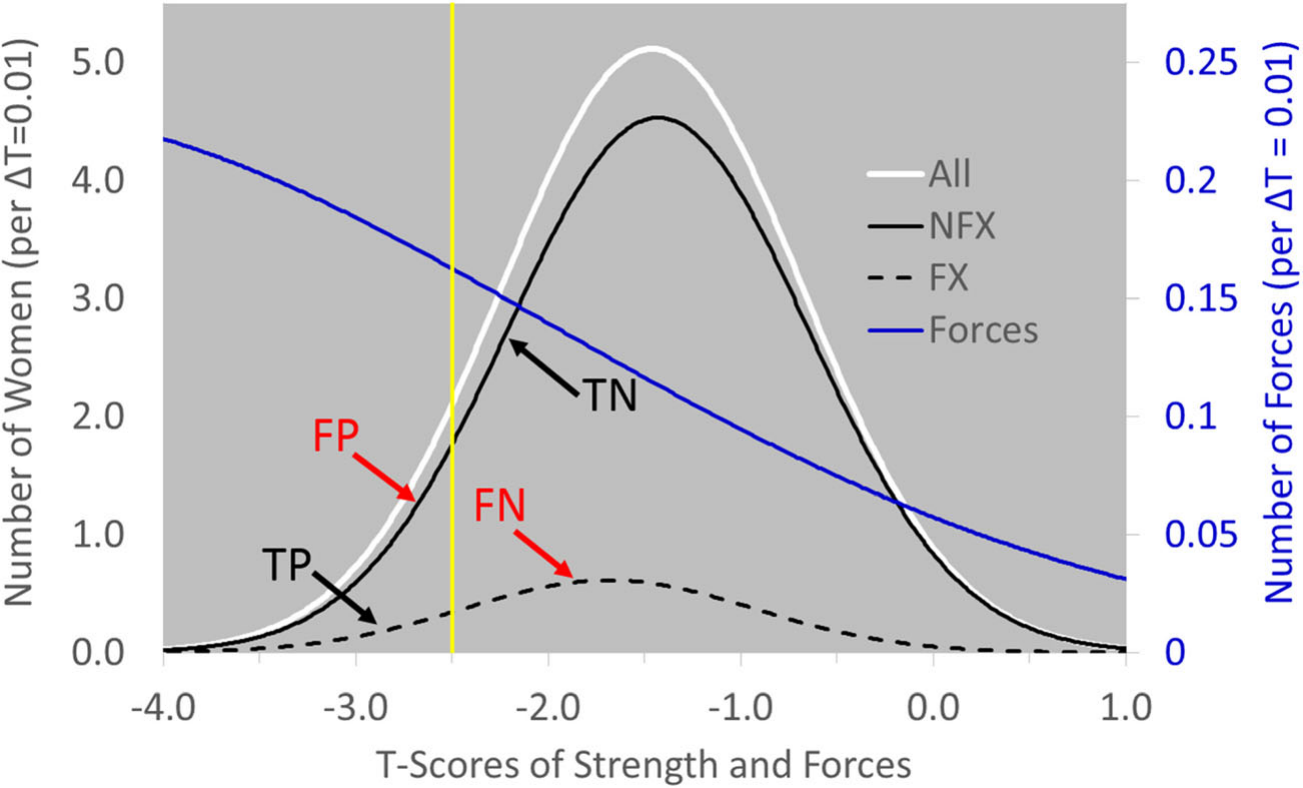
Hypothetical distributions of strength in women with and without fractures exposed to a skewed distribution of forces. (All: white solid line = all women; NFX: black solid line = women with fractures, FX: black broken line = women without fractures; Forces: blue solid line; treatment threshold: yellow solid line at T_Strength_ = −2.5; TP = True Positive, FP = False Positive, FN = False Negative, TN = True Negative)

Now suppose the women in Fig. 2 come from a single normal distribution of bone *strength* instead of aBMD T-scores (All in Fig. 5 below); and suppose the distribution of forces acting upon them every day is skewed, with small forces outnumbering large ones (Forces in Fig. 5). Then multiplying the two distributions generates the same FX and NFX distributions as those in Fig. 4! Moreover, in Fig. 5, FN do not have undetected weak bone, as in Fig. 3. *FN have strong bone and are numerous in* Fig. 5*only because of the law of large numbers: the large number of women above the diagnostic threshold of bone strength overwhelms the rarity of the large forces that act upon them.*

Thus, the Heavy Load Hypothesis infers from Fig. 5 that FN in Fig. 2 did *not* fracture because they had weak bones. Rather, they fell or were otherwise impacted by large forces [17]. If the Heavy Load Hypothesis is true, a more effective strategy for reducing FPR and FNR would be to identify patients with poor neuro-motor function and to treat *them* to prevent falls [18]: dance class and table tennis, perhaps, instead of calcium and bisphosphonates. *Moreover, if the Heavy Load Hypothesis is true, then treatment of patients with more accurately estimated weak bone would not improve FPR and FNR, because FP would continue to be treated and FN would continue to go untreated.*

If an accurate method for measuring bone strength was to emerge; however, one of the first things to do with it would be to test the Weak Bone and Heavy Load Hypotheses. In science, hypotheses are tested by falsifying them. Accordingly, if patients were to be allocated to treatment by T_aBMD_ < −2.5 and treatment were to be withheld from those with T_aBMD_ > −2.5 as in current practice and:

- If the new, more accurate method were to find bone in FN to be s*tronger* than bone in FP, then the Weak Bone Hypothesis would be false.
- If the new, more accurate method were to find bone in FN to be w*eaker* than bone in FP, then the Heavy Load Hypothesis would be false.

### Cortical Bone Mechanics Technology™ (CBMT)

Unlike the monotonically increasing displacement imposed directly onto a bone in ex vivo quasistatic single load-to-failure tests, CBMT imposes only a small static force (10–20 *N* = 2–4 pounds) and an even smaller oscillatory force (1 *N* = 3 oz) to the skin overlying the mid-shaft of the ulna bone. The static load is like the force felt on a fingertip when pressing an elevator button, and the oscillatory force is like the vibration one feels when holding an electric toothbrush. The oscillations range in frequency from 40 to 1200 Hz.

**Fig. 6:**
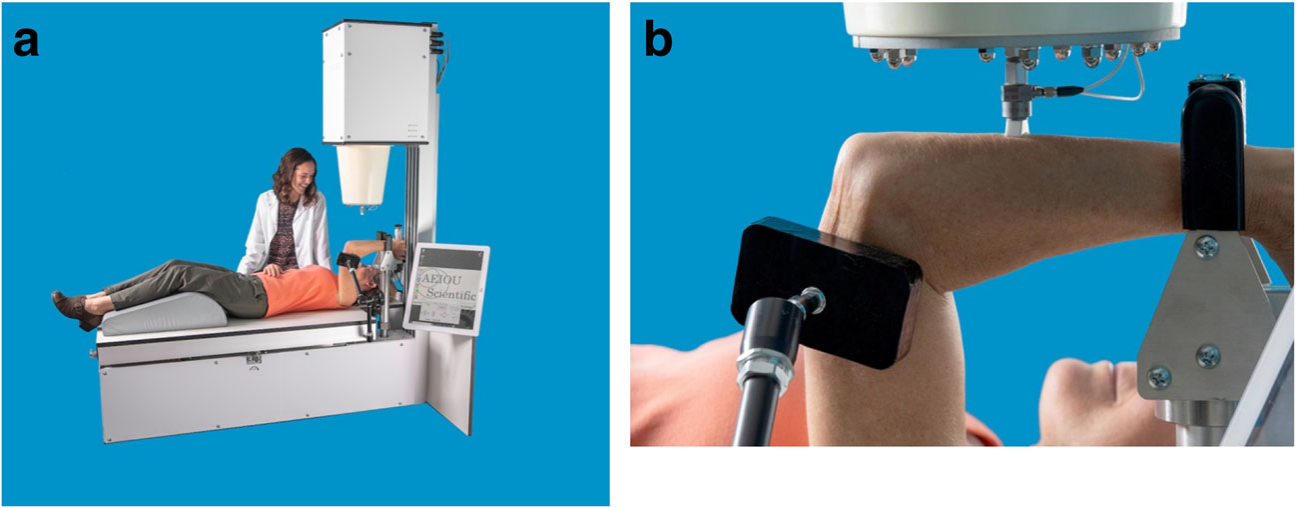
Wide angle (**a**) and close-up (**b**) views of a CBMT test in progress. The ulna is tested because, of all the bones in the body, the biomechanics of the ulna are the most ideal for a bending test in vivo.

A CBMT test is a *functional* test in that it measures how the ulna actually behaves mechanically (i.e., bends) under an applied load. Figure 6 shows a CBMT test in progress. The operator positions the subject for testing and then starts a robot that lowers and lifts the probe, collects and analyzes data under controlled loading conditions at various sites across the ulna, and reports results immediately. The test takes 15–20 min to complete.

The ulna is tested because, of all the bones in the body, the biomechanics of the ulna are the most ideal for a bending test in vivo. As shown in the close-up view in Fig. 6, the ulna can be positioned with its proximal end supported at the trochlear notch by the articulating trochlea of the humerus and with its distal end supported by a rigid platform via the styloid process of the radius. No other long bone in the body can be so supported. A bending test is performed to ensure that the measured bone tissue is unambiguously cortical: a bending test is especially sensitive to mechanical properties at the mid-shaft of a long bone, where bone tissue is entirely cortical. Any apparently trabecular bone at the mid-shaft of long bones is actually “trabecularized” residual cortical bone between intra-cortical resorption spaces. By contrast, the distinction between cortical and trabecular bone at the distal ends of long bones is ambiguous [19].

Force and acceleration data are analyzed as a frequency response function that is fitted to a 7-parameter mathematical model of the mechanical skin-bone system to quantify the stiffness, damping and mass of the ulna and its overlying skin, as well as the damping of the surrounding soft tissue that the bone pushes aside as it vibrates. Because comparisons of ulna bending stiffness (k) are confounded by differences in ulna length (L), ulna bending stiffness is converted to ulna flexural rigidity (EI = kL^3^/48) by Euler-Bernoulli beam theory [20] to enable ulnas of different people to be compared to one another. EI is the product of the elastic modulus (E) of ulna bone *material* and the cross-sectional moment of inertia (I) of ulna bone *geometry* in antero-posterior bending. It is to be emphasized that EI is thereby derived as a *product* without separate determination of E or I. Thus, the values of E and I implicit in EI are both *patient-specific* rather than assumed a priori or derived from imagery of bone mineral.

In the above calculations, CBMT is identical to a previous method known as Mechanical Response Tissue Analysis (MRTA) [20]. MRTA was developed by Charles Steele at Stanford University in the 1980s, patented by Stanford, and licensed for commercialization. Commercialization was abandoned in the 1990s when the adoption of DXA for diagnosing osteoporosis eliminated the incentive and investment available for further development. Recently, Ohio University patented novel methods described elsewhere [3•, 21, 22•] for correcting sources of error in MRTA caused by its high sensitivity to probe positioning and by the impossibility of an operator identifying the correct place to position the probe by sight or touch. That problem is overcome by artificial intelligence in the CBMT robot derived from extensive studies of cadaveric human arms. DXA performance data such as those in Fig. 2 enabled the novelties in CBMT to be licensed for commercialization (www.aeiouscientific.com).

#### Validation

**Fig. 7:**
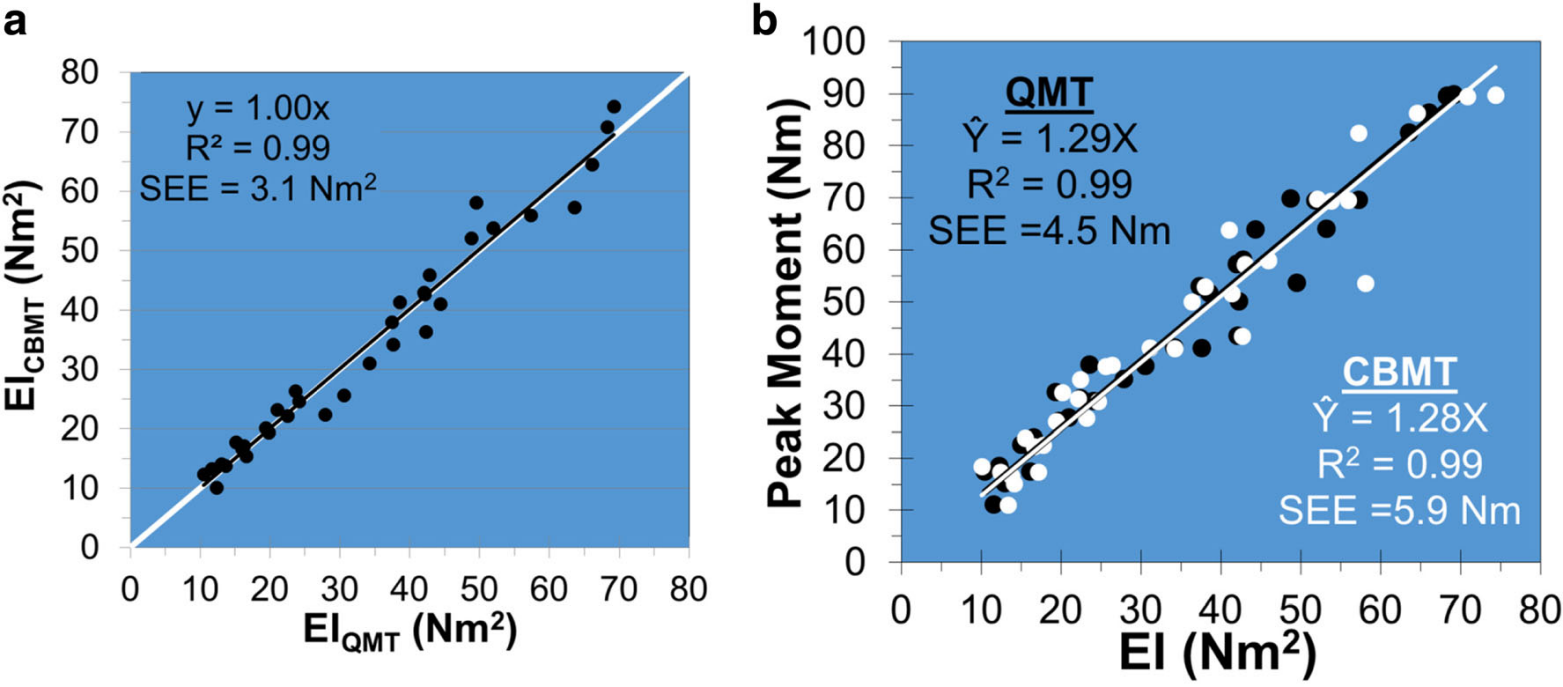
CBMT technical validity (**a**): ulna flexural rigidity (EI) calculated from CBMT and quasistatic (QMT) measurements of ulna bending stiffness. CBMT clinical validity (**b**): ulna bending strength (peak moment) measured by QMT in excised ulnas in relation to ulna EI calculated from CBMT and QMT measurements of ulna bending stiffness. ( [3•], used with permission from Elsevier)

Because bone strength can only be measured ex vivo, the accuracy of any method for estimating bone strength must be performed on cadaveric tissue. Figure 7 (left) shows the near identity (R^2^ = 0.99) of ulna flexural rigidity (EI) calculated from noninvasive CBMT and ex vivo quasistatic single load-to-failure (QMT) tests of ulna bending stiffness in 35 cadaveric human arms from male and female donors varying widely in age (17 to 99 years), height (1.47 to 1.88 m; i.e., 4 ft. 10 in to 6 ft. 2 in), and body mass index (13 to 40 kg/m^2^) [3•]. Figure 7 (right) shows the accuracy (R^2^ = 0.99) of CBMT and QMT estimations of ulna bending strength (Ŷ) by ulna EI**.** The estimations were indistinguishable from one another (*p* > 0.80). Figure 7 (left) is evidence of the *technical validity* of CBMT (i.e., CBMT measures what it purports to measure), while Fig. 7 (right) is evidence of its *clinical validity* (i.e., CBMT measurements reflect the clinical condition of the patient: ulna bending strength).

#### Total Error Rate

Figure 7 (right) can be used to compare the total error rate (TER) of CBMT to that of other indirect methods for estimating direct QMT measures of bone strength for which corresponding graphs have been published. Table 1 compares TER values for estimations of ulna bending strength by CBMT measurements of ulna EI and by microtomographic measurements of cortical porosity [3•] as well as TER values for estimations of femoral neck bending strength by femoral neck aBMD T-scores, tibial cortical porosity and tibial indentation (Biodent™; Active Life Sciences, Santa Barbara, CA) [23].

**Table 1:** Total error rate (TER) for estimations of ulna and femoral neck bending strength indirectly by EI (flexural rigidity), aBMD T-scores (T_aBMD_), cortical porosity (CP), and indentation (IDI)

Reference	Bone (n)	Predictor	SEE (Nm)	${\hat{Y}}_{max}$-${\hat{Y}}_{min}$ (Nm)	TER (%)
[3•]	Ulna (35)	EI	5.9	82.2	7%
		CP	18.9	55.8	34%
			SEE (N)	${\hat{Y}}_{max}$-${\hat{Y}}_{min}$ (N)	
[23]	Femoral neck (28)	T_aBMD_	1291	6476	20%
		Tibia CP	1823	6146	44%
		Tibia IDI	1837	4337	42%

The high TER values for predictions of ulna and femoral neck bending strength by cortical porosity in Table 1 are consistent with high error rates (FPR = 75%, FNR = 67%) in allocations of patients to treatment by cortical porosity [24]. Combining cortical porosity with aBMD T-scores improved but did not solve the problem (FPR = 72%, FNR = 53%) [24].

Four models of reference point indentation devices have been produced over the years (Biodent™, Osteoprobe I™, Osteoprobe II™ and Osteoprobe®; Active Life Sciences, Santa Barbara, CA), but the empirical basis of all of them is measurement of an increment of indentation by a stylus into the periosteal surface of a bone relative to one reference or another [25]. In general, indentation techniques measure hardness, which is *not* a mechanical property but rather a *relationship* between two materials. Differences in bone hardness have been detected between patients with and without various clinical conditions affecting bone health — sometimes [26–29], but the mechanical properties affecting indentation of bone are obscure [25, 30–32]. No significant association was found between indentation measured by the Osteoprobe® and any mechanical property of bone [32], and the strongest association reported between indentation measured by the Biodent™ and any mechanical property of bone (R^2^ = 0.33) was with the toughness of hydrated cortical bone specimens [31, 33]. As toughness is a *material* rather than structural mechanical property, the technique is now promoted for assessing cortical bone at the hierarchical level of bone *tissue* [34]. In contrast to indentation, the *structural* mechanical properties measured by CBMT (ulna stiffness, damping, and mass) are clear, and they characterize the mechanical behavior of a whole bone. *The clinical utility of CBMT* (i.e., *whether its lower TER in predicting ulna bending strength improves the targeting of treatment at FN instead of FP and thereby patient outcomes) has not yet been tested.*

#### Dynamic Mechanical Loading

**Fig. 8:**
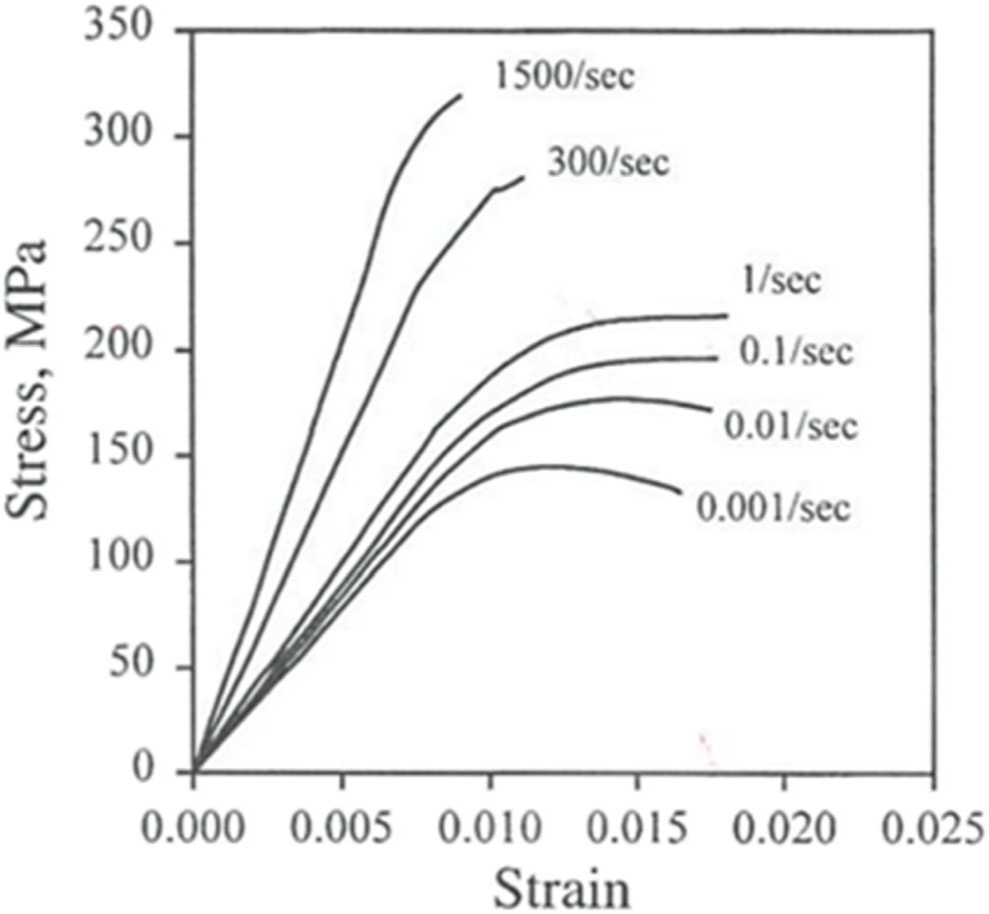
Stress-strain curves for bovine bone specimens loaded at various constant strain rates (from [35] redrawn with permission from [36]). Used with permission from Springer Nature

When a person falls from a standing height, his hip hits the ground at up to 10 miles per hour. That is like running at a 6 min/mile pace — into a wall. Figure 8 shows stress-strain curves from single load-to-failure tests of bovine bone specimens under quasistatic (strain rate έ = 0.001/s) and dynamic (i.e., higher strain rate) conditions. Note that the strength (i.e., peak *stress* before fracture) of bone *tissue* is much greater under dynamic loads than quasistatic ones. The following discussion explains why.

Under the dynamic conditions of a fall or collision, the relationship between force and motion in the linear elastic region of bone displacement is:

$$ \mathrm{F}=\mathrm{kx}+\mathrm{dv}+\mathrm{ma} $$

where F is the force applied to the bone; where x, v, and a are resulting displacement, velocity, and acceleration of the bone; and where k, d, and m are bone’s stiffness, damping, and mass. By contrast, under unrealistic, quasistatic conditions of a single load-to-failure test, x is made to increase so slowly that v ≈ a ≈ 0 so that the relationship between force and motion reduces to:

$$ \mathrm{F}=\mathrm{kx} $$

This enables the bone’s stiffness to be measured as the slope of the force-displacement curve.

A bone can support much higher loads without breaking under dynamic conditions, because the higher loads are shared by the bone’s viscous (dv) and inertial (ma) as well as elastic (kx) load carrying capacities. (The constant strain rates in Fig. 8 elicited only viscous and elastic effects.) Thus, bone damping and mass are protective against fracture under dynamic loads. *Bone damping measured in our weakest cadaveric ulnas would have increased their strength by 40% in a 5 mph collision.*

Bone damping is a property of bone protein, and the elevated fracture risk in patients with Type 2 diabetes is attributed to the decoration of collagen with advanced glycation end products (AGEs) [37]. In our study of cadaveric arms, CBMT measurements of ulna damping were 25% lower in ulnas of nine donors with Type 2 diabetes than in ulnas of the same size in arms of donors without diabetes (*p* = 0.03). *The technical validity of CBMT measurements of ulna damping and mass has not yet been confirmed by independent measures in dynamic single load-to-failure tests. Nor has the clinical validity of those measurements been demonstrated by accurate estimates of ulna bending strength under the dynamic conditions of a fall or collision. Whether such clinically validated estimates are clinically useful for improving FPR and FNR also remains to be investigated.*

## Conclusion

Direct measurement of the mechanical properties of cortical bone in vivo may prove to be clinically useful in the assessment and monitoring of bone fragility in many clinical conditions besides osteoporosis associated with aging (including chronic kidney disease, HIV, cancer, cystic fibrosis, rheumatoid arthritis, bariatric surgery, spinal cord injury, androgen deprivation, corticosteroid treatment, anorexia nervosa, and the female athlete triad) as well as other systemic bone diseases (including osteogenesis imperfecta, osteomalacia, Paget’s disease, hypophosphatasia, and hyperparathyroidism) and constitutional thinness. The commercially available CBMT scientific instrument shown in Fig. 6 is intended for use by scientists in studies of cortical bone mechanics in such human subjects. Its technical and clinical validity under quasistatic conditions have been demonstrated. Its validity under dynamic conditions remains to be determined. The CBMT scientific instrument is not a medical device and is not intended for use by health-care professionals to diagnose, monitor, or treat disease. For that purpose, an investigational CBMT medical device is under development. Its clinical utility has yet to be tested.
